# Capillary Self-Alignment of Microchips on Soft Substrates

**DOI:** 10.3390/mi7030041

**Published:** 2016-03-04

**Authors:** Bo Chang, Quan Zhou, Zhigang Wu, Zhenhua Liu, Robin H. A. Ras, Klas Hjort

**Affiliations:** 1 Department of Engineering Sciences, Uppsala University, SE-75121 Uppsala, Sweden; zhigang.wu@angstrom.uu.se (Z.W.); klas.hjort@angstrom.uu.se (K.H.); 2 Department of Applied Physics, School of Science, Aalto University, FI-00076 Aalto, Finland; robin.ras@aalto.fi; 3 Department of Electrical Engineering and Automation, Aalto University, FI-00076 Aalto, Finland; quan.zhou@aalto.fi; 4 State Key Laboratory of Digital Manufacturing Equipment and Technology, Huazhong University of Science and Technology, Wuhan 430074, China; liuzhenhuahust@qq.com

**Keywords:** capillary self-alignment, soft micro devices, stretchable electronics, superhydrophobic PDMS, hydrophilic/superhydrophobic patterned surfaces

## Abstract

Soft micro devices and stretchable electronics have attracted great interest for their potential applications in sensory skins and wearable bio-integrated devices. One of the most important steps in building printed circuits is the alignment of assembled micro objects. Previously, the capillary self-alignment of microchips driven by surface tension effects has been shown to be able to achieve high-throughput and high-precision in the integration of micro parts on rigid hydrophilic/superhydrophobic patterned surfaces. In this paper, the self-alignment of microchips on a patterned soft and stretchable substrate, which consists of hydrophilic pads surrounded by a superhydrophobic polydimethylsiloxane (PDMS) background, is demonstrated for the first time. A simple process has been developed for making superhydrophobic soft surface by replicating nanostructures of black silicon onto a PDMS surface. Different kinds of PDMS have been investigated, and the parameters for fabricating superhydrophobic PDMS have been optimized. A self-alignment strategy has been proposed that can result in reliable self-alignment on a soft PDMS substrate. Our results show that capillary self-alignment has great potential for building soft printed circuits.

## 1. Introduction

Soft micro devices have attracted great interest for their potential applications in sensory skins and wearable bio-integrated devices. Just like in rigid systems, one path to achieving larger systems or systems at lower volumes at reasonable costs is to use printed circuit technology. To allow for stretchable printed circuit boards, the alignment of assembled micro sized components is an important step, where it normally requires precise positioning of the micro parts. However, to use common pick and place technology on surfaces that are skewed and stretched poses great difficulty in pattern recognition and positioning, which reduces throughput. One solution to reducing the demand of position accuracy is to let the components self-align.

Capillary self-alignment is based on the principle of minimum potential energy, where the gradient of the potential is designed to drive the parts towards desired locations. The key for successful capillary self-alignment is to confine the droplet inside the patterns. One promising approach toward preventing liquid spreading is to use a patterned surface where a large wetting contrast between patterns and their surrounding is preferred. Capillary self-alignment has been reported as achieving high-precision assembly on patterned oleophilic/superoleophobic surfaces [1], protruding patterns [2,3], hydrophilic/hydrophobic patterned surfaces [4,5,6,7,8,9], hydrophilic/superhydrophobic patterned surfaces [10,11] and all-hydrophobic patterns [12]. Moreover, high yield self-assembly has been reported in water [13]. In a recent industrial demonstration, a throughput of over 40,000 units per hour and micron accuracy positioning has been demonstrated [14]. Previously, capillary self-alignment has also been applied to flexible materials [15,16,17]. However, it is an open question if it is possible to achieve capillary self-alignment of chips on soft materials. To study this, we first need to create hydrophilic-hydrophobic patterns on a soft material, and understand the influence of the softness of the material to self-alignment parameters and processes.

In this work, we fabricated patterned soft surfaces that consist of flat hydrophilic pads surrounded by superhydrophobic PDMS surrounding. Using such a patterned PDMS substrate, we studied the capillary self-alignment and noticed that the system behaves differently in the alignment process compared to studied cases on rigid substrates. Here, we have provided a novel self-alignment strategy for the reliable alignment of microchips on soft PDMS substrates.

## 2. Experimental Section

**Figure 1:**
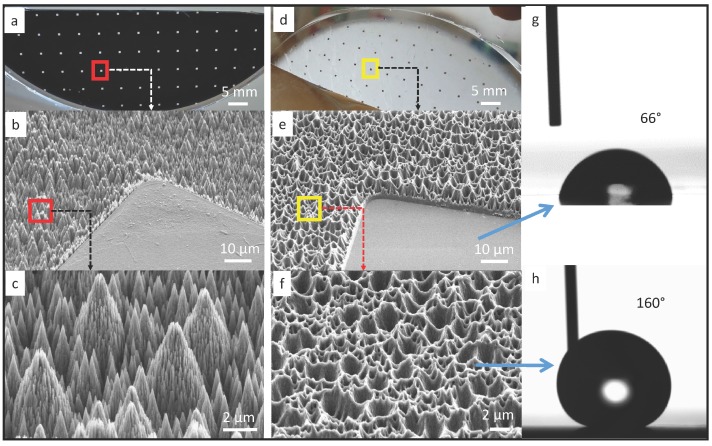
Patterned black silicon surface and PDMS replicate: (**a**) 500 µm × 500 µm SiO_2_ pads surrounded by black silicon surface coated with fluoropolymer, with scanning electron micrograph in (**b**) and (**c**); (**d**) PDMS replicate with scanning electron micrograph in (**e**) and (**f**); (**g**) static water contact angle of 66° on the flat pad and (**h**) 160° on the nanostructured Trichlorosilane treated PDMS surface.

In our work, soft substrate is fabricated using polydimethylsiloxane (PDMS), which is one of the most commonly used materials in microfluidics [18] and stretchable electronics [19,20] due to its high compliance and biocompatibility. To achieve capillary self-alignment, it is essential to have a large wetting contrast between the pads and their surrounding [21]. Furthermore, based on our previous work, a superhydrophobic surrounding surface could largely increase the reliability and tolerance of the self-alignment [11]. Therefore, we have developed a simple stamping process for making superhydrophobic patterned PDMS surfaces. A patterned black silicon sample was prepared as a master, and PDMS was used as a stamp to replicate the micro- and nanostructures from the black silicon master. The patterned black silicon was fabricated according to a procedure reported in our previous work [10]. The fabricated master consists of 500 µm × 500 µm SiO_2_ square pads surrounded by the black silicon background coated with fluoropolymer (see Figure 1a). The measured static water contact angle on the SiO_2_ pad and black silicon substrate are 50° and 170°, respectively. The combination of the nanostructures on the black silicon and the low surface energy fluoropolymer coating are the main contributors for the superhydrophobicity of the substrate. To make superhydrophobic patterned PDMS surfaces, first, a PDMS mixture was prepared by mixing the base and curing agent at a ratio of 10:1, and the mixture was then degassed in a vacuum desiccator for 10 min to remove the air bubbles. Next, the mixture was poured over the black silicon master and baked in an oven at 75 °C for 90 min. After that, the cured PDMS was peeled off from the master and then functionalized with Trichloro (1H,1H,2H,2H-perfluorooctyl) silane (Sigma-Aldrich, St. Louis, Mo, USA) inside a vacuum desiccator for 12 h. Silane is a commonly used reagent in surface science to form a self-assembled monolayer for decreasing the surface energy. Scanning electron micrographs of the fabricated black silicon master and the replicated PDMS stamp are seen in Figure 1b–f. The surface of the black silicon master has pine-tree like structures (see Figure 1c), which consist of micro-scale cone structures and an over layer of short nano-scale needle-like structures. The structures on the black silicon master have been successfully replicated into the PDMS surface (see Figure 1e,f). Although the structures on the PDMS surface are the negative of the black silicon master, the hydrophobicity of the PDMS surface has been largely enhanced, as demonstrated in the measured static water contact angle (see Figure 1h). The measured static water contact angle on the flat pad is 66° (Figure 1g), while the water contact angle on the Trichlorosilane treated superhydrophobic PDMS surface is 160°. The large water contact angle indicates that the water droplet is in Cassie state on the PDMS surface with replicated micro- and nanostructures. The combination of the large wetting contrast and the superhydrophobicity of the PDMS surrounding are key to confining the liquid inside the pad, which is important for successful and reliable capillary self-alignment.

**Figure 2:**
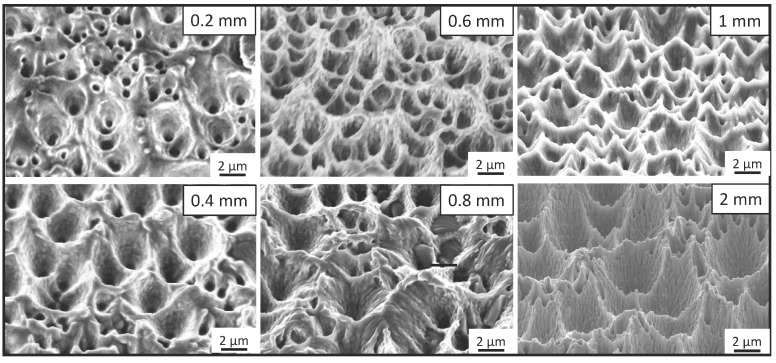
Scanning electron micrographs of PDMS substrates with thickness of 0.2 mm, 0.6 mm, 0.4 mm, 0.8 mm, 1 mm and 2 mm, indicating that the thicker the PDMS substrate is, the more detailed features able to be replicated are.

The parameters used in the PDMS replication process have been optimized by investigating a commonly used PDMS (Sylgard 184) with the base-to-agent mixing ratio of 10:1 and 15:1. The results indicate that the Sylgard 184 can be used to replicate micro- and nanostructures from black silicon. However, with the mixing ratio of 10:1, the PDMS substrate can be easily peeled off. With the base to curing agent ratio of 15:1, the compliance of the cured PDMS becomes much lower, which makes the peeling-off from the black silicon master very difficult. Another important parameter is the thickness of the PDMS substrate, which has a clear influence on the replicated micro structures. We fabricated PDMS stamps with different thicknesses from 0.2 mm to 2 mm, and the results are presented in Figure 2.

When the substrate is 0.2 mm thick, only part of the cone structures on the black silicon master are successfully replicated onto the PDMS stamp, and the needle-like nanostructures are not able to be replicated. As the thickness of the PDMS stamp increases, more structures can be replicated, as visualized in Figure 2. The reason for the imperfect replication on the thinner stamp could be that the thinner the stamp is, the shorter the curing time is; combined with less pressure by its mass, it does not allow for sufficient contact between the stamp and the black silicon structures.

**Figure 3:**
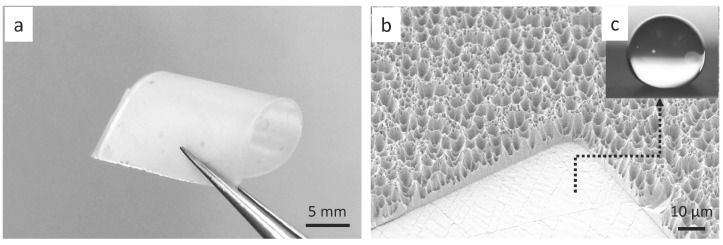
Superhydrophobic PDMS stamp with Pd pads: (**a**) a soft patterned PDMS substrate; (**b**) scanning electron micrograph of a corner of a Pd pad with PDMS substrate; (**c**) a droplet of water is confined inside a Pd pad surrounded by superhydrophobic PDMS substrate.

Since electrical contacts in stretchable electronics are normally made of metal materials, we have fabricated metal pads on the PDMS surface. The superhydrophopic PDMS stamp was prepared using the same replication procedure introduced above. After the stamp was made, the sample surface was treated with plasma for improved adhesion; then, the sample was covered by a shadow mask and coated with 40 nm of Pd via sputtering. Using the same method, it was also possible to pattern the superhydrophobic PDMS with other metals such as Au or Al. The shadow mask was prepared by using a tape plotter and aligned so that Pd was only deposited on the flat pads. The fabricated superhydrophobic PDMS substrate with Pd pads is shown in Figure 3a, and the microstructures are seen in the scanning electron micrograph (Figure 3b). When a droplet of water was deposited on a Pd pad, the droplet was able to be confined inside the flat Pd pad (see Figure 3c). The accuracy of the Pd pad is mainly depending on the accuracy of the shadow mask, which is about 10 µm.

## 3. Results and Discussion

**Figure 4:**
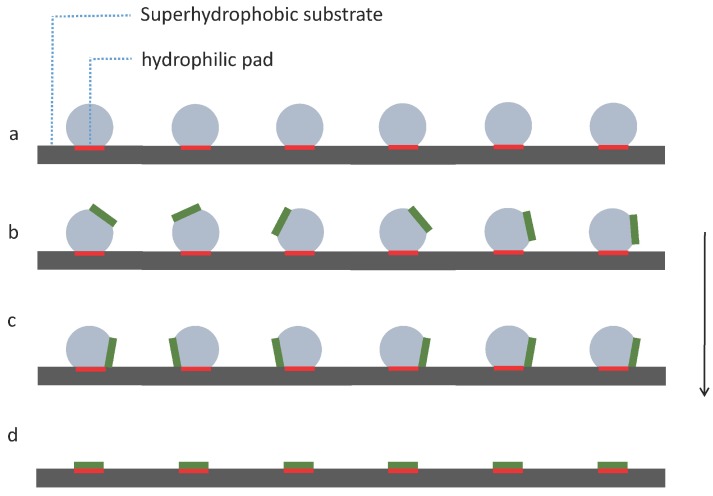
Capillary self-alignment strategy on a softsubstrate: (**a**) Droplets are deposited on patterned hydrophilic/superhydrophobic PDMS surface; (**b**) chips are randomly placed on the droplets; (**c**) droplets are evaporating and chips are tilted; (**d**) droplets are evaporated and chips (in green) are aligned with the pads (in red).

We propose a simple strategy as shown in Figure 4 to investigate capillary self-alignment on superhydrophobic patterned PDMS substrates. The droplets were deposited on hydrophilic pads using a liquid dispenser, and each droplet was well confined inside the corresponding pad due to the large wetting contrast between the hydrophilic pad and its superhydrophobic PDMS surrounding (Figure 4a); next, micro-sized chips were placed randomly on the droplets (Figure 4b); the chips then either slid to the right or to the left due to the unbalanced forces (Figure 4c); then, the droplets started evaporating, and finally disappeared leaving the chips (in green) aligned with the corresponding pads (in red) (Figure 4d). The superhydrophobicity of the substrate plays an important role in the strategy we proposed here, because the strong dewetting behavior of the superhydrophobic substrate prevents droplet spreading outside the pad and ensures reliable self-alignment.

**Figure 5:**
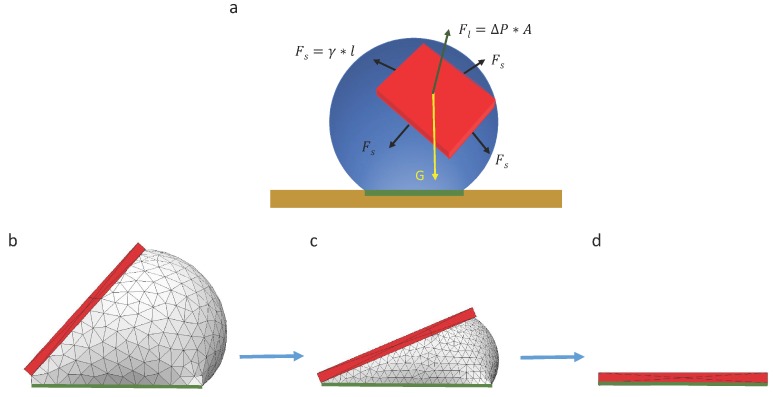
Analytical analysis and numerical simulation of capillary self-alignment: (**a**) The force of a chip floating on a droplet is configured; (**b**) a chip is tilted and floating on a droplet; (**c**) the droplet gradually evaporates and the chip tilts accordingly; (**d**) the droplet evaporates leaving the chip (in red) aligned with the pad (in green).

When a chip is randomly placed on a droplet, there are three forces acting on the chip, including gravitational force G, surface tension force $F_{s}$ along the triple contact line and the surface tension force $F_{l}$ caused by Laplace pressure, as illustrated in Figure 5. Surface tension force along the triple contact line can be described as $F_{s}=\gamma *l$, where l is the width of the chip, and γ is the surface tension of the liquid per unit length. The surface tension caused by Laplace pressure can be calculated as $F_{l}=\Delta P*A$, where ΔP is the pressure difference caused by the surface tension of the interface between liquid and air, and A is the area of the chip. Since the droplet is symmetric, the Laplace pressure can be described as ΔP=4γ/d, where d is the diameter of the droplet. In our experiments, the size of the pad was 500 µm × 500 µm, which is much smaller than the capillary length of water (2.7 mm); therefore, we believe the capillary force is more dominant than the gravity of the chip during the whole self-alignment process.

Due to the unbalanced gravitational force and capillary force at the beginning of the capillary self-alignment process, the chip quickly slides downwards. Then, the chip reaches a quasi-static state where the component of the surface tension force and gravitational force are balanced. After that, the droplet starts evaporating, and this process can be numerically simulated with Surface Evolver [22] (see Figure 5b–d), which breaks the surface into smaller elements and minimizes the surface energy by optimizing the location of each vertex on the droplet. The surface energy of the droplet during its evaporation process can be calculated if the volume of the droplet is known at each quasi-static state. The simulation result indicates that the surface energy of the droplet is minimized when the chip is perfectly aligned with the pad (see Figure 5d).

**Figure 6:**
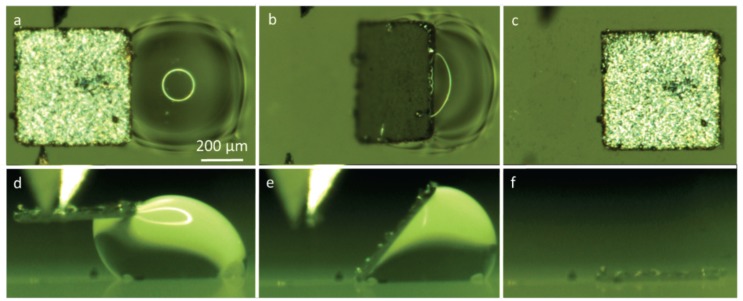
Top-view and side-view images of capillary self-alignment of a 500 µm × 500 µm chip on a superhydrophobic patterned PDMS substrate (extracted from Supplementary Videos S1 and S2): (**a**,**d**) a chip was ready to be released with extreme large placement bias (470 µm); (**b**,**e**) once the chip was released by a gripper, the chip was tilted and one edge was aligned to the edge of the pad; (**c**,**f**) the droplet evaporated and the chip was aligned with the pad.

A series of experiments were conducted to study capillary self-alignment on soft substrates. The proposed handling strategy was implemented on PDMS patterned surfaces which consisted of 500 µm × 500 µm pads surrounded by a superhydrophobic PDMS substrate. The measured water contact angle on the pad and the substrate were 66° and 160°, respectively. The microchips were fabricated by cutting a 30-µm thick stainless steel sheet using micro-laser technology and the microchips had the same dimension as the pads. A liquid dispenser was used to dispense water droplet on the pad, and a custom-made piezo actuated microgripper was used to place microchips on the water droplets. We investigated the influence of the volume of the droplet and the placement bias on the capillary self-alignment process. The volume of the droplet was in the range from 5 nL to 360 nL, and the placement bias varied from 50 µm to 500 µm. The placement bias is defined as the distance between the center of the chip and the pad. When the bias was larger than the size of the pad, it means that the chip was placed outside the pad. Figure 6 shows an example of capillary self-alignment with a placement bias of 470 µm in top view (a–c) and side view (d–f) (the images were extracted from Supplementary Videos S1 and S2). A chip was moved towards the pad and in contact with the droplet (Figure 6a,d); the chip slid down and one edge was aligned to the edge of the pad immediately after the chip was released from the gripper (Figure 6b,e); and the self-alignment was achieved as the water droplet evaporated. A successful self-alignment is defined as no visible placement error observed under the optical microscope.

The result matches well with the simulation shown in Figure 5b–d and indicates that the process can tolerate extremely large placement bias. The volume of the droplet is 60 nL, and the capillary self-alignment takes about 130 s. Self-alignment tests with different volumes of the droplet (5 to 360 nL) were carried out to find out the boundary conditions, and each test was repeated five times. The results are summarized in Table 1. It shows that the self-alignment process is very robust to the volume of the droplet and self-alignment is reliable with a droplet volume ranging from 10 to 360 nL. It is a big improvement compared to our earlier studies [9] showing that the volume of the droplet has to be carefully controlled for successful self-alignment using hydrophilic/hydrophobic patterned surfaces. With the volume of the droplet less than 10 nL, self-alignment starts to fail mainly because there is not enough liquid to cover the pad, which leads to dry contact and large friction force between the partially wetted pad and the chip. Additionally, the volume of the droplet does have influence on the duration of the self-alignment. More liquid leads to a longer evaporation time and therefore a longer self-alignment time.

We tested different placement biases ranging from 50 µm to 500 µm, the volume of the droplet was kept the same (60 nL) for all the tests, and each test was repeated five times. The results indicate that the bias has little influence on the capillary self-alignment process regarding the self-alignment speed and self-alignment yield. Table 2 summarizes the results.

**Figure 7:**
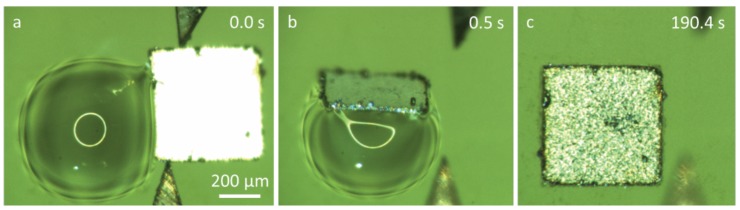
Capillary self-alignment of a 500 µm × 500 µm chip with 510 µm placement bias: (**a**) A chip was ready to be released from the gripper; (**b**) the chip slid down and was aligned to the edge of the pad; (**c**) the droplet evaporated and the chip was aligned with the pad.

We also studied an extreme case of capillary self-alignment with a placement bias larger than the size of the pad and with a droplet volume of 90 nL. Figure 7 shows a case where the chip was released outside the pad (Figure 7a) and successful self-alignment was achieved. The result indicates that the superhydrophobic patterned surface can largely enhance the tolerance of the capillary self-alignment process to placement error, which agrees with a recent report on a rigid superhydrophobic patterned black silicon substrate [11]. It is interesting to note that the microchip has rotated about 90° against the center of the droplet, and continued the self-alignment process from the top side instead of the initial right side where the chip was placed onto the droplet. This also indicates that the chip is self-aligning to the pad at a very early stage of the process due to the dominant surface tension.

In comparison, we have also investigated self-alignment using water mist, which has been used in previous reports of self-alignment [11]. The chip was firstly placed on the top of a pad with initial placement bias, and microscopic droplets in the form of water mist were then introduced for self-alignment. Unlike the previous reported results, where the chip was able to self-align with the pad surrounded by the superhydrophobic black silicon background with extreme large placement bias [11], our result shows that the chip failed to align with the pad with a superhydrophobic PDMS substrate. The failed self-alignment is very likely caused by the relatively large adhesion force on the soft PDMS surface, associated with the softness of the material.

## 4. Conclusions

In summary, we have developed a simple fabrication process for making superhydrophobic patterned PDMS surfaces and proposed a novel strategy for self-alignment of rigid components on a soft PDMS substrate that can result in successful self-alignment. Despite the slight differences in the self-alignment process compared to more widely used processes on rigid or flexible substrates, we demonstrate that the self-alignment of chips on a soft substrate is viable and reliable. This may lead to a high-throughput assembly of chips in soft micro devices and stretchable printed circuit boards.

## Figures and Tables

**Table 1:** Influence of volume of droplet on capillary self-alignment.

Volume (nL)	Success Rate	Self-Alignment Duration (s)
360	100%	451
150	100%	235
60	100%	132
20	100%	55
10	80%	38
5	0%	N.A.

**Table 2:** Influence of placement bias on capillary self-alignment.

Placement Bias (µm)	Success Rate	Self-Alignment Duration (s)
50	100%	125
100	100%	132
200	100%	135
300	100%	124
400	100%	130
500	100%	127

## Supplementary Materials

The following are available online at http://www.mdpi.com/2072-666X/7/3/41/s1. Video S1: Top view of the capillary self-alignment on a hydrophilic/superhydrophobic patterned PDMS surface. Video S2: Side view of the capillary self-alignment on a hydrophilic/superhydrophobic patterned PDMS surface.
